# Enhancing Metabolic Imaging of Energy Metabolism in Traumatic Brain Injury Using Hyperpolarized [1-^13^C]Pyruvate and Dichloroacetate

**DOI:** 10.3390/metabo11060335

**Published:** 2021-05-24

**Authors:** Stephen J. DeVience, Xin Lu, Julie L. Proctor, Parisa Rangghran, Juliana A. Medina, Elias R. Melhem, Rao P. Gullapalli, Gary Fiskum, Dirk Mayer

**Affiliations:** 1 Department of Diagnostic Radiology and Nuclear Medicine, University of Maryland School of Medicine, Baltimore, MD 21201, USA; stephen@scalarmag.com (S.J.D.); xin-lu@ouhsc.edu (X.L.); emelhem@umm.edu (E.R.M.); rgullapalli@som.umaryland.edu (R.P.G.); 2 Center for Metabolic Imaging & Therapeutics (CMIT), University of Maryland Medical Center, Baltimore, MD 21201, USA; 3 Department of Anesthesiology and the Center for Shock, Trauma, and Anesthesiology Research (S.T.A.R.), University of Maryland School of Medicine, Baltimore, MD 21201, USA; jproctor@som.umaryland.edu (J.L.P.); prangghran@som.umaryland.edu (P.R.); jmedina@som.umaryland.edu (J.A.M.); gfiskum@som.umaryland.edu (G.F.)

**Keywords:** traumatic brain injury, magnetic resonance spectroscopic imaging, hyperpolarized metabolic imaging, pyruvate dehydrogenase, controlled cortical impact

## Abstract

Hyperpolarized magnetic resonance spectroscopic imaging (MRSI) of [1-^13^C]pyruvate metabolism has previously been used to assess the effects of traumatic brain injury (TBI) in rats. Here, we show that MRSI can be used in conjunction with dichloroacetate to measure the phosphorylation state of pyruvate dehydrogenase (PDH) following mild-to-moderate TBI, and that measurements can be repeated in a longitudinal study to monitor the course of injury progression and recovery. We found that the level of ^13^C-bicarbonate and the bicarbonate-to-lactate ratio decreased on the injured side of the brain four hours after injury and continued to decrease through day 7. Levels recovered to normal by day 28. Measurements following dichloroacetate administration showed that PDH was inhibited equally by PDH kinase (PDK) on both sides of the brain. Therefore, the decrease in aerobic metabolism is not due to inhibition by PDK.

## 1. Introduction

Traumatic brain injury (TBI) is a leading cause of death and disability in people under the age of 45 and can lead to lifelong cognitive impairment [1,2,3]. TBI is known to cause perturbations in the energy metabolism of the brain, which may be linked to injury severity and progression [4]. In particular, the replacement of aerobic respiration with anaerobic metabolism might have important consequences for patient management and ultimate outcome. However, traditional in vitro assessments of metabolism, such as cerebral blood flow and metabolite measurements [5], as well as microdialysis [5,6,7], are highly invasive and difficult to use in a clinical setting. A less invasive method, ^18^F-fluorodeoxy-glucose positron emission tomography (FDG PET) [8], provides only indirect measurements of metabolic activity.

Recently, works by ourselves and others have shown that magnetic resonance spectroscopy (MRS) of hyperpolarized [1-^13^C]pyruvate can be used to evaluate TBI non-invasively in mice and rats [9,10]. MRS enables real-time imaging of metabolic activity with a single MRI scan by measuring the conversion of ^13^C-labeled pyruvate into lactate and bicarbonate. Following TBI, the resulting ^13^C-bicarbonate signal was found to be lower on the injured side of the brain, indicating decreased pyruvate dehydrogenase (PDH) activity. This loss of activity could be due to cell death [11], enzyme loss [12,13], enzyme dysfunction, or enzyme inhibition by a number of mechanisms, including phosphorylation by PDH kinase (PDK).

To further explore whether PDK inhibition plays a role in TBI, in this work, we present additional hyperpolarized MRS experiments of TBI using [1-^13^C]pyruvate following the administration of dichloroacetate (DCA), a PDK inhibitor. This removes the effect of PDK so that the uninhibited state of PDH can be probed [14]. We found that DCA roughly doubled the bicarbonate signals on both sides of the brain, but the bicarbonate signal remained lower on the injured side, indicating that inhibition by PDK is unlikely to be a major influence. The non-invasive nature of MRS also makes it a convenient way to monitor the time course of injury and recovery. We therefore expanded on our previous study by performing longitudinal measurements on additional days 2, 7, and 28 after the TBI. We found that bicarbonate and the bicarbonate-to-lactate ratio (bic/lac ratio) continued to decrease on the injured side of the brain through day 7 but returned to normal levels by day 28.

## 2. Results

Results for day 0, measured 4 h after CCI injury, were reported previously [9]. To summarize, we acquired traditional T_2_-weighted fast-spin echo (FSE) images to evaluate the area of injury and to select the proper slice for hyperpolarized ^13^C imaging. The image typically showed hyperintensity in the cerebral cortex at the CCI location, and some rats also exhibited swelling in the area around the injury. The hyperpolarized MRSI images showed a clear difference between control and injured animals. Images of control animals, both visually and quantitatively, showed symmetric metabolite signals across both sides of the brain, whereas images of injured animals showed symmetric pyruvate signals but a lower bicarbonate signal, a higher lactate signal, and a lower bic/lac ratio on the ipsilateral side. Following injury, the bicarbonate signal was 24 ± 6% lower on the injured side of the brain, and the bic/lac ratio was 33 ± 8% lower versus controls.

**Figure 1:**
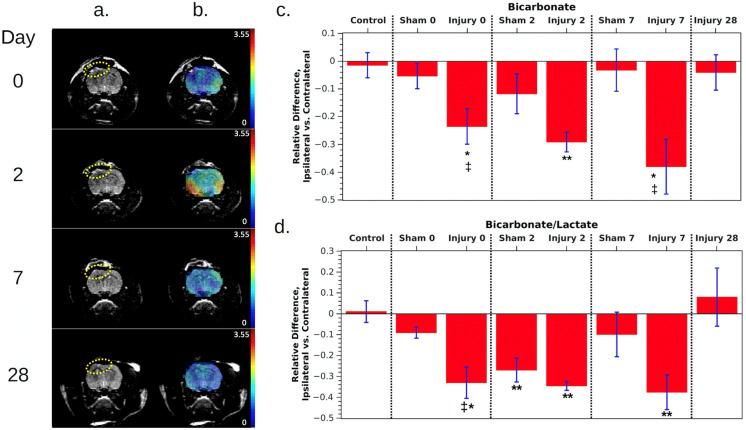
(**a**) T_2_-weighted fast spin echo images of a rat following CCI (left hemisphere of the image, indicated by the dotted ellipses). The windowing level was adjusted to emphasize the hyperintense region of injury. (**b**) The same images with an overlay of the bicarbonate maps. (**c**,**d**) Relative difference in metabolites between ipsilateral and contralateral ROIs, averaged over all rats measured at each time point. Individual measurements are shown by black symbols, and each rat has been assigned its own symbol. Also see Table 1 in the methods for the number of rats in each group. All error bars are standard error. Red* indicates that *p* < 0.05 compared with control animals, ** indicates *p* < 0.01 compared with controls, and ‡ indicates *p* < 0.05 compared with shams.

The same measurements were repeated on days 2, 7, and 28 after the injury. Figure 1 shows the overall time course for FSE and bicarbonate images from an animal following CCI, as well as the mean differences of integrated signals for bicarbonate and bic/lac for the whole study cohort. In the FSE images, the hyperintense area at the site of injury remained consistent between day 0 and 7 but decreased to a smaller size by day 28. The bicarbonate images also show less intensity beneath the site of injury than on the contralateral side. The relative difference in the bicarbonate signal between ipsi- and contralateral sides decreased further during the first week, from 24 ± 6% at day 0 to 38 ± 10% at day 7, and the difference was statistically significant on days 0, 2, and 7 compared with controls (*p* = 0.02, 0.0004, and 0.03, respectively). The bic/lac ratio also decreased, becoming 42 ± 7% lower on the ipsilateral side by day 7, and the results were also statistically significant for all three days 0, 2, and 7 compared with controls (*p* = 0.004, 0.0001, and 0.008, respectively). However, by day 28, both parameters had returned to normal.

Measurements of animals given sham surgeries showed roughly the same pattern, but the milder injury resulted in a smaller decrease in bicarbonate and in the bic/lac ratio on the injured side, reaching a 12 ± 7% and 27 ± 6% decrease, respectively, on day 2. The effect only reached statistical significance for the bic/lac ratio on day 2 compared with controls (*p* = 0.0070). The metabolite levels nearly returned to normal by day 7, and sham data were not acquired on day 28. Unlike CCI animals, where bicarbonate and bic/lac decreased roughly the same amount, for shams there was a stronger decrease in the bic/lac ratio. This may be due to partial volume effects causing lactate signal in the inflamed region around the brain to leak into the brain ROI, skewing the measurement. Swelling and inflammation tended to be most obvious in T_2_-weighted images on day 2.

**Figure 2:**
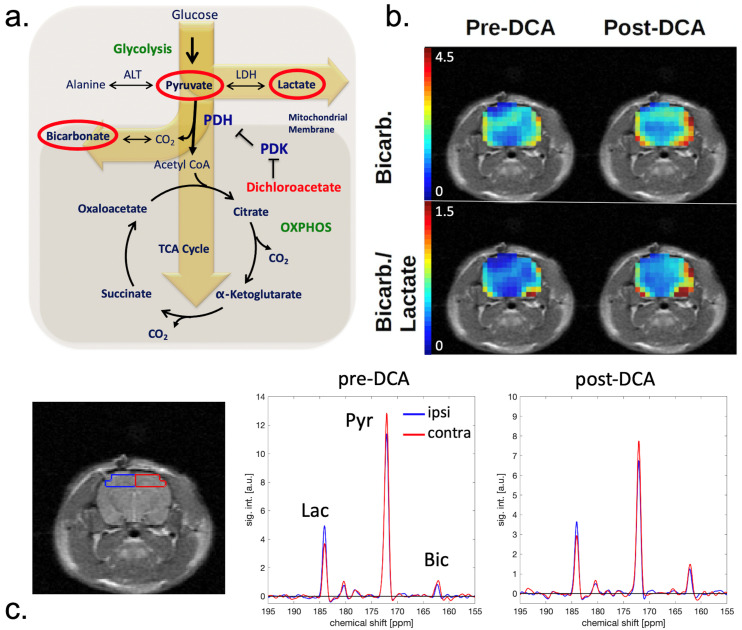
(**a**) Schematic of the tricarboxylic acid cycle. [1-^13^C]pyruvate is converted to [1-^13^C]lactate via lactate dehydrogenase (LDH) and to ^13^C-bicarbonate via pyruvate dehydrogenase (PDH). PDH is inhibited by pyruvate dehydrogenase kinase (PDK), which in turn is inhibited by dichloroacetate (DCA). The overall effect of DCA is to increase PDH activity. (**b**) Bicarbonate and bic/lac images of a CCI rat on day 0 before and after DCA administration. The intensity scale is the same for pre- and post-DCA maps. (**c**) Set of corresponding spectra acquired from ROIs in the ipsilateral (blue) and contralateral (red) hemisphere. The location of the ROIs is indicated on the corresponding MRI (left).

**Figure 3:**
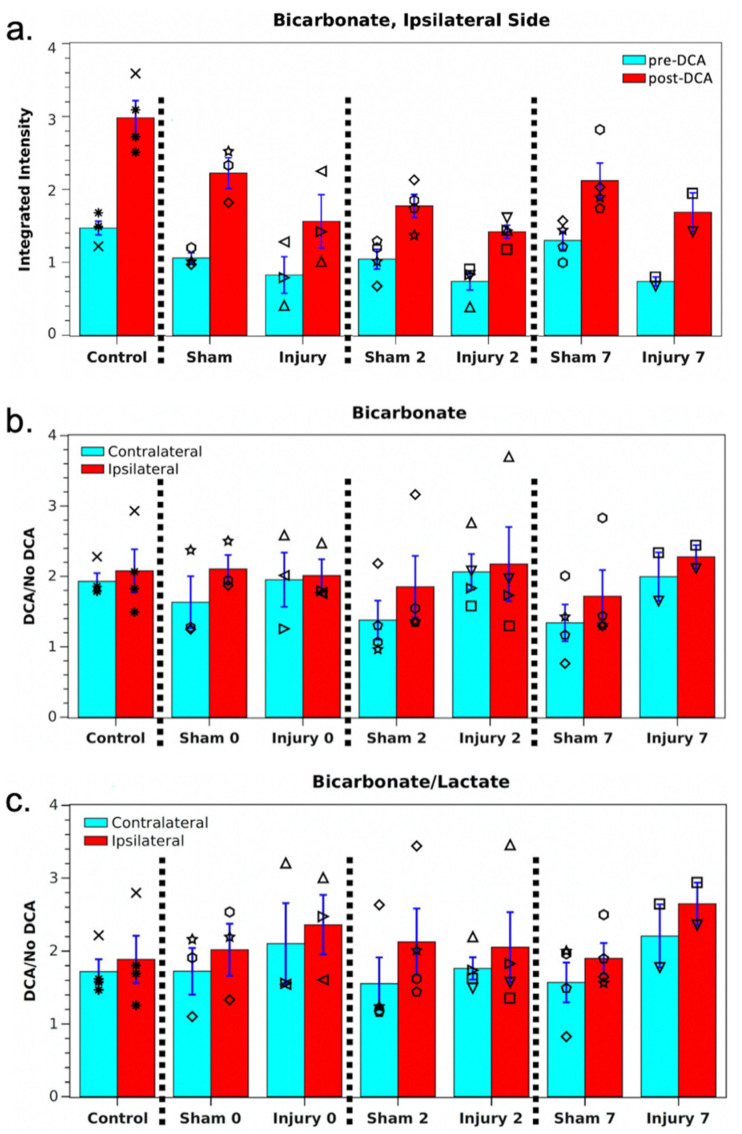
(**a**) Mean ipsilateral bicarbonate for the cohort pre- and post-DCA. Individual measurements are shown by black symbols, and each rat has been assigned its own symbol. (**b**,**c**) Mean bicarbonate and bic/lac signal enhancements for the cohort following DCA administration. None of the measurements showed a significant difference between hemispheres or versus controls.

At each session, hyperpolarized images were also acquired 45 min after DCA administration. These showed no significant differences in pyruvate or lactate signals compared with pre-DCA images. However, as shown in Figure 2; Figure 3, the average bicarbonate signal was significantly stronger following DCA administration. The integrated bicarbonate signal roughly doubled on both sides of the brain, as did the bic/lac ratio. This relative increase in the bicarbonate signal was not significantly different between ipsilateral and contralateral sides, so the measurement continued to show a lower overall level of bicarbonate on the injured side. Neither bicarbonate nor the bic/lac ratio returned to control levels. The same effect was seen on days 0, 2, and 7. Metabolic maps for pyruvate, lac, and bicarbonate from a single animal pre- and post-DCA at the four time-points are shown in Supplemental Figure S1.

## 3. Discussion

Since the detection and measurement of metabolic changes following traumatic brain injury is important for patient management, a non-invasive technique that can be repeated regularly for follow-up, such as hyperpolarized ^13^C MRSI, is advantageous compared with invasive methods such as microdialysis. We were able to reliably acquire images on multiple days following TBI in rats, and thereby track the course of injury progression and healing.

The longitudinal results suggest that, in rats, traumatic brain injury causes significant metabolic dysfunction, beginning at the time of injury and extending for at least a week afterward. The decrease in both the raw bicarbonate signal and in the bic/lac ratio on the injured side indicates a drop in aerobic metabolism. Sham animals also exhibit a lesser but similar trend, suggesting that the surgery itself causes mild alterations in local metabolism. By day 28, the injury appears to have healed enough that the bicarbonate and bic/lac values return to normal. This is also reflected by a decrease in the hyperintense region in T_2_-weighted images. The timeline of injury and recovery is in agreement with previous studies using different MRI metrics. For example, Long et al. found that T_2_ was maximum between 3 h and 2 days after injury, then trended toward normal values between days 2 and 14 [15]. Apparent diffusion coefficient peaked at day 2 and returned to normal by day 14. Wang et al. found a nearly identical time course for mean diffusivity [16].

The decrease in PDH activity observed following injury could be caused by several different mechanisms. One possibility is that the enzyme activity is inhibited by loss of the PDH cofactor, thiamine pyrophosphate [17]. Since PDH activity is also inhibited by pyruvate dehydrogenase kinase (PDK) and by the NADH/NAD+ and acetyl-CoA/CoA ratios, dysregulation of any of these biomolecules could be responsible [18,19]. Another possibility is that loss of enzyme activity is due to protein modifications, such as those generated by reactions with reactive oxygen and nitrogen species [20]. It is also likely that such mechanisms of inactivation may be dissimilar at different times after injury [21]. Dichloroacetate inhibits PDH kinase, thereby stimulating PDH activity. This effect has been measured previously both via chemical assay [22,23] and hyperpolarized imaging [14]. Administration of DCA to the rats after TBI resulted in an enhancement of bicarbonate labeling; however, enhancement was the same for the ipsilateral and contralateral sides. The conclusion is that PDH is inhibited by PDK equally on both sides. Decreased PDH activity on the injured side must therefore be due to one of the other mechanisms, e.g., oxidative protein alterations.

The results of this study support the idea that decreased metabolic activity is due to the drop in the PDH concentration following TBI that has been measured by a number of groups. For example, Sharma et al. found a significant decrease in brain PDH following whole-brain TBI, as measured by a dipstick antibody test [12]. Xing et al. also found a significant decrease in PDH protein, as well as PDH mRNA, following CCI but, in their case, the protein concentration was lower on both sides of the brain, not just the injured side [13]. However, further experiments are needed to rule out effects of NADH or acetyl-CoA concentrations. In addition to probing the effects of PDH inhibition, administration of DCA could be exploited to enhance the bicarbonate signal in MRSI of hyperpolarized [1-^13^C]pyruvate without skewing the relative balance of glycolytic and oxidative metabolism between injured and normal tissue. This is advantageous, as the bicarbonate signal is usually much smaller than lactate. The higher SNR could then be leveraged to increase the spatial resolution to reduce partial volume effects and better localize the injury. DCA has also been studied as a treatment for some types of brain injury and brain tumor, and hyperpolarized imaging might be helpful in evaluating its effectiveness [22,23].

There are several limitations to the present study and to others measuring PDH activity following traumatic brain injury. The first is that the CCI model is one of several that are used routinely to study mild to severe injury. The use of other models, e.g., the lateral fluid percussion model, should be used to confirm the general applicability of our conclusions. The same is true for lab animal species. Rats and mice are the species used most typically in animal models of brain injury; however, other species, e.g., pigs and ferrets, are being used increasingly due to the similarity of their gyrencephalic brains in comparison to human neuroanatomy. We also only used adult male rats in the present study. Future studies should determine whether the effects of head trauma and associated loss of PDH activity are different between sexes [24] and with age [21].

## 4. Materials and Methods

### 4.1. Controlled Cortical Impact (CCI)

Six male rats (five Sprague-Dawley and one Wistar, with weights ranging from 230 to 260 g) were used to induce CCI injury. Animals were anesthetized with isoflurane/O_2_ at 1 mL/min (3% for induction, 2% for maintenance), and placed on a homeothermic heating pad to maintain body temperature. Buprenorphine was given for analgesia. Animals were head-fixed in a stereotactic apparatus and a left-hemisphere craniotomy was performed using a surgical bone micro-drill at approximately 3.5 mm AP and 4 mm ML to bregma [25]. After removing the bone flap and exposing the dura, rats were positioned in the CCI device. To cause a mild-to-moderate TBI [26], the exposed area was struck with a 5 mm diameter beveled flat impactor tip at a velocity of 5 m/s and a 50-ms impact duration, resulting in a deformation depth of 2.0 mm. Immediately after CCI, the bone flap was replaced, sealed with dental acrylic, the scalp was sutured shut, and animals were allowed to recover. For sham surgeries (four male Sprague-Dawley rats), all the steps listed above were taken, but no CCI was delivered.

### 4.2. Animal Handling during Magnetic Resonance Imaging (MRI)

At the start of the imaging session, rats were anesthetized as described above and a 24 G tail vein catheter was placed. In the MRI magnet the animals received anesthesia via a nose cone while a pad with circling warm water was used to regulate body temperature.

### 4.3. Polarization and Injection

Hyperpolarized [1-^13^C]pyruvate (~135 mM at pH ~7) was generated using a 5 T GE SPINlab (Research Circle Technology, Niskayuna, NY, USA) as previously described [9]. The pyruvate solution was manually injected via the tail vein catheter within 30 s of dissolution over a duration of ~12 s at a dose of 1.1 mmol/kg.

### 4.4. MR Acquisitions

A 3 T GE 750w scanner (GE Healthcare, Waukesha, WI, USA) and a dual-tuned ^13^C/^1^H quadrature coil (50 mm diameter, USA Instruments Inc., Aurora, OH, USA) were used for the experiments. T_2_-weighted fast spin echo (FSE) ^1^H-MRI was performed to locate the site of the injury. 2D-MRSI of a single 8 mm slice in the coronal plane (transaxial to the magnet system, 2.5 mm nominal resolution) centered at the injury was acquired 30 s after start of the pyruvate injection as described before [27].

### 4.5. Dichloroacetate

A subset of animals was imaged after an acute injection of dichloroacetate. Following the first MRSI acquisition on the respective day, the rat remained under anesthesia and was injected with a dose of dichloroacetate (200 mg/kg, 30 mg/mL saline) via the tail vein. After 45 min, hyperpolarized ^13^C MRSI was performed again in an identical fashion to the first acquisition.

### 4.6. Longitudinal Study

The first ^13^C MRSI image was acquired three to four hours after CCI injury. Identical experiments were performed 2, 7, and 28 days later on surviving rats. Not all rats survived the full length of the study.

Identical control experiments were performed on nine male Sprague-Dawley and two male Wistar rats either two days prior to injury or on age-matched individuals with no injury. These were performed only once and not repeated for days 2, 7, and 28. Identical experiments were also performed on four male Sprague-Dawley rats on which sham surgeries were performed. These rats underwent a left-sided craniotomy, but the bone flap was replaced and sealed without the application of a CCI injury. The control experiments were performed on days 0, 2, and 7. Table 1 summarizes the number of rats in each group.

### 4.7. Data Processing

The ^13^C MRSI data were processed using custom written MATLAB-based software, as described before [9], comprising apodization (Hanning window in spatial k-space and 25-Hz Gaussian window in spectral dimension) and zero-filling (2-fold in both spatial dimensions and 8-fold in the spectral dimension). Metabolic maps were calculated by integrating the respective metabolite peak in absorption mode [28].

For assessment of altered metabolism, the spectra from a region of interest (ROI) within injured hemisphere (encompassing the neocortex and the parts of the hippocampus) were averaged, and the respective signal intensities for pyruvate, lactate, and bicarbonate were calculated by fitting a Gaussian function to the respective peak in absorption mode. For display of individual spectra shown in Figure 2 and Supplementary Figure S3, a first-order phase correction was applied with the baseline subtracted by fitting a spline to signal-free regions of the spectrum. Spectra from an ROI in the contralateral hemisphere were evaluated in the same manner for comparison. Examples for size and location of the ROIs are shown in Figure 2 and Supplementary Figure S3. Metabolite ratios for each ROI were computed as the ratio of integrated signals. These data were then used to compare the injured and non-injured hemispheres. To quantify the ipsilateral vs. contralateral signal difference within each rat, the relative difference Δ was calculated:

Δ = [(ipsilateral) − (contralateral)]/(contralateral)

Statistical significance between control and injury groups, as well as between ipsilateral and contralateral signals, was calculated using a two-tailed Welch’s *t*-test for independent samples. Ipsilateral and contralateral signals were also compared using a two-tailed *t*-test for correlated samples. For the longitudinal study, a two-tailed Welch’s *t*-test for independent samples was used to compare the ipsi/contra ratio on each day following TBI with that day’s shams as well as with the controls. All results and plots show (mean) ± (standard error). A value of *p* < 0.05 was considered significant.

## Figures and Tables

**Table 1:** Number of rats imaged on each day after CCI injury or sham surgery, pre- and post-DCA. For those animals that were imaged post-DCA, the compound was administered acutely, i.e., during the respective imaging session.

Group	Day 0		Day 2		Day 7		Day 28	
DCA	Pre	Post	Pre	Post	Pre	Post	Pre	Post
Control	11	4	-	-	-	-	-	-
Sham	4	3	4	4	4	4	0	0
CCI	6	3	4	4	4	2	4	2

## Data Availability

The data presented in this study are available from the corresponding author, D.M., upon reasonable request.

## Supplementary Materials

The following are available online at https://www.mdpi.com/article/10.3390/metabo11060335/s1, Figure S1: Longitudinal pyruvate, lactate, and bicarbonate images in a CCI rat before and after DCA administration, The same rat was imaged on days 0 through 28, No DCA was administered on day 0, The intensity scale in the metabolic maps is the same across time points, Figure S2: Longitudinal images of bicarbonate/lactate ratio and lactate/bicarbonate ratio in a CCI rat before and after DCA administration, The same rat was imaged on days 0 through 28, No DCA was administered on day 0, The intensity scale in the ratio maps is the same across time points, Figure S3: Longitudinal set of spectra in a CCI rat from ROIs in the ipsilateral (blue) and contralateral (red) hemisphere, The location of the ROIs is indicated on the corresponding MRIs (left), Spectra are shown both before (middle) and after (right) DCA administration, The same rat was imaged on days 0 through 28, No DCA was administered on day 0, Table S1: Results of Welch’s *t*-test for four different comparisons of bicarbonate concentration and bicarbonate-to-lactate ratio.
