# Supplementary material for: Enhancing Metabolic Imaging of Energy Metabolism in Traumatic Brain Injury Using Hyperpolarized [1-13C]Pyruvate and Dichloroacetate
Source: Metabolites. 2021 May 24;11(6):335. doi: 10.3390/metabo11060335 (PMC8225170; doi:10.3390/metabo11060335)
Supplement: Supplementary file 1 [file metabolites-11-00335-s001.zip › suppl/Supp_Material.pdf]

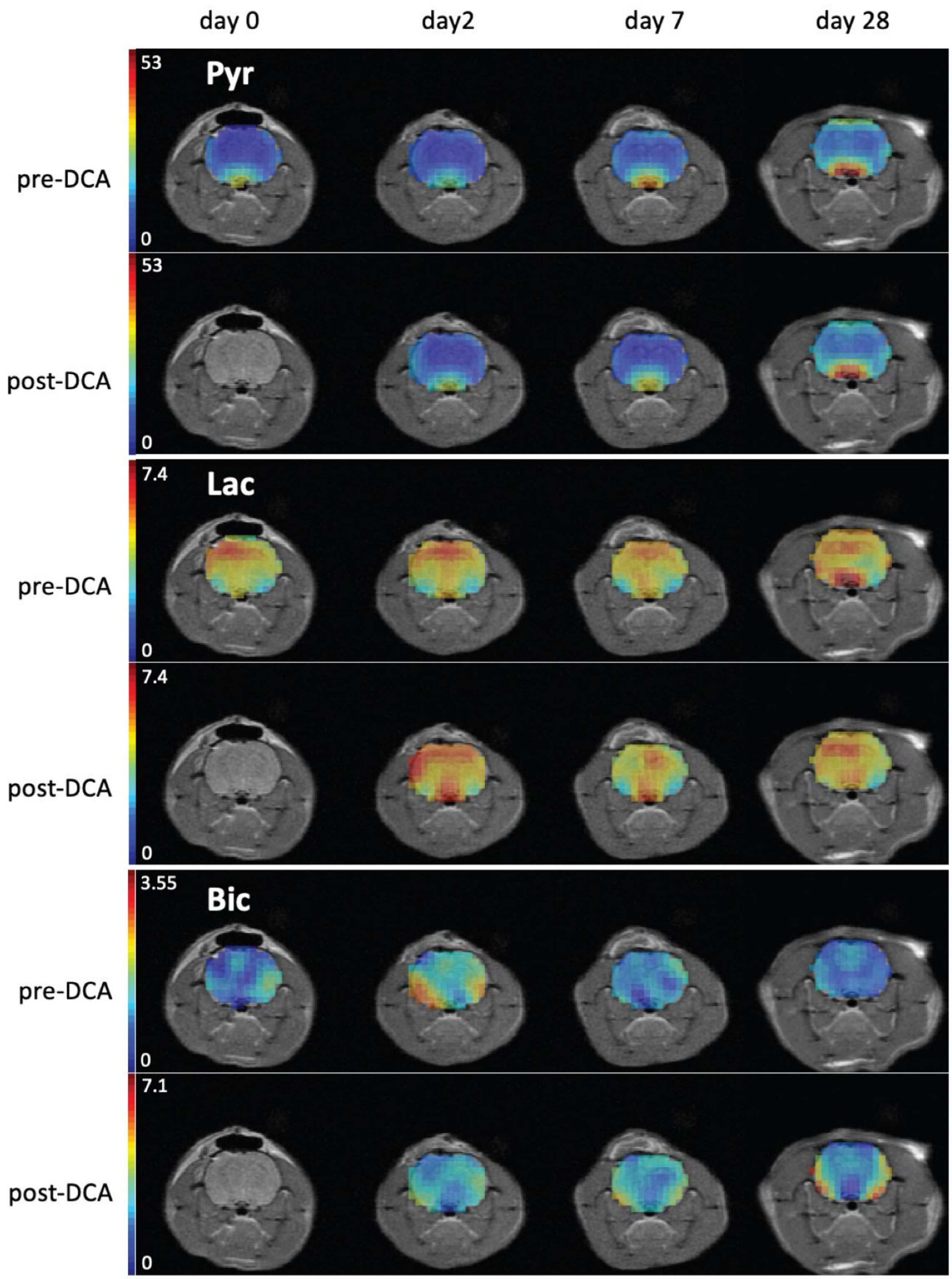

Figure 1. Longitudinal pyruvate, lactate, and bicarbonate images in a CCI rat before and after DCA administration. The same rat was imaged on days 0 through 28. No DCA was administered on day 0. The intensity scale in the metabolic maps is the same across time points.

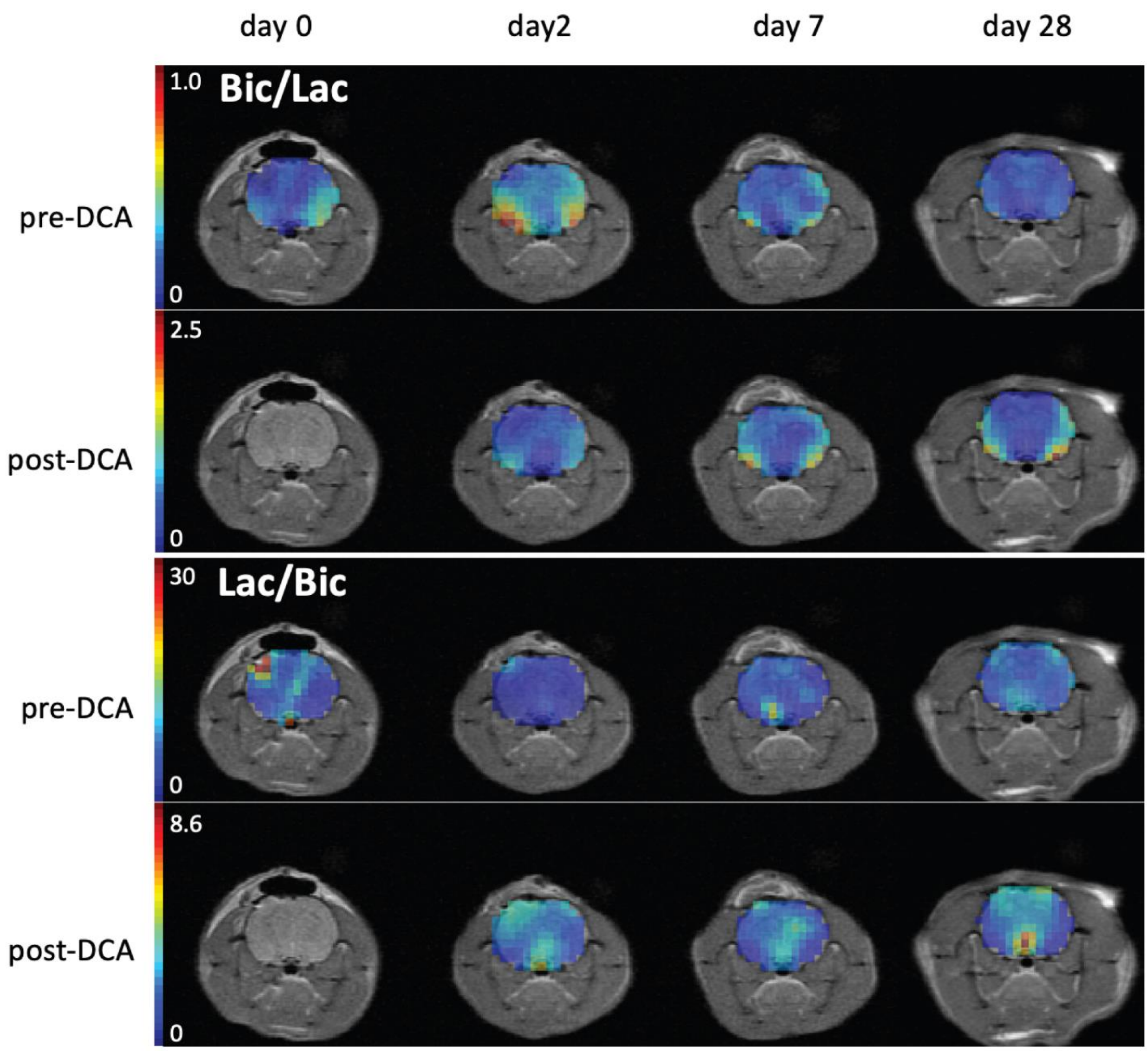

Figure S2. Longitudinal images of bicarbonate/lactate ratio and lactate/bicarbonate ratio in a CCI rat before and after DCA administration. The same rat was imaged on days 0 through 28. No DCA was administered on day 0. The intensity scale in the ratio maps is the same across time points.

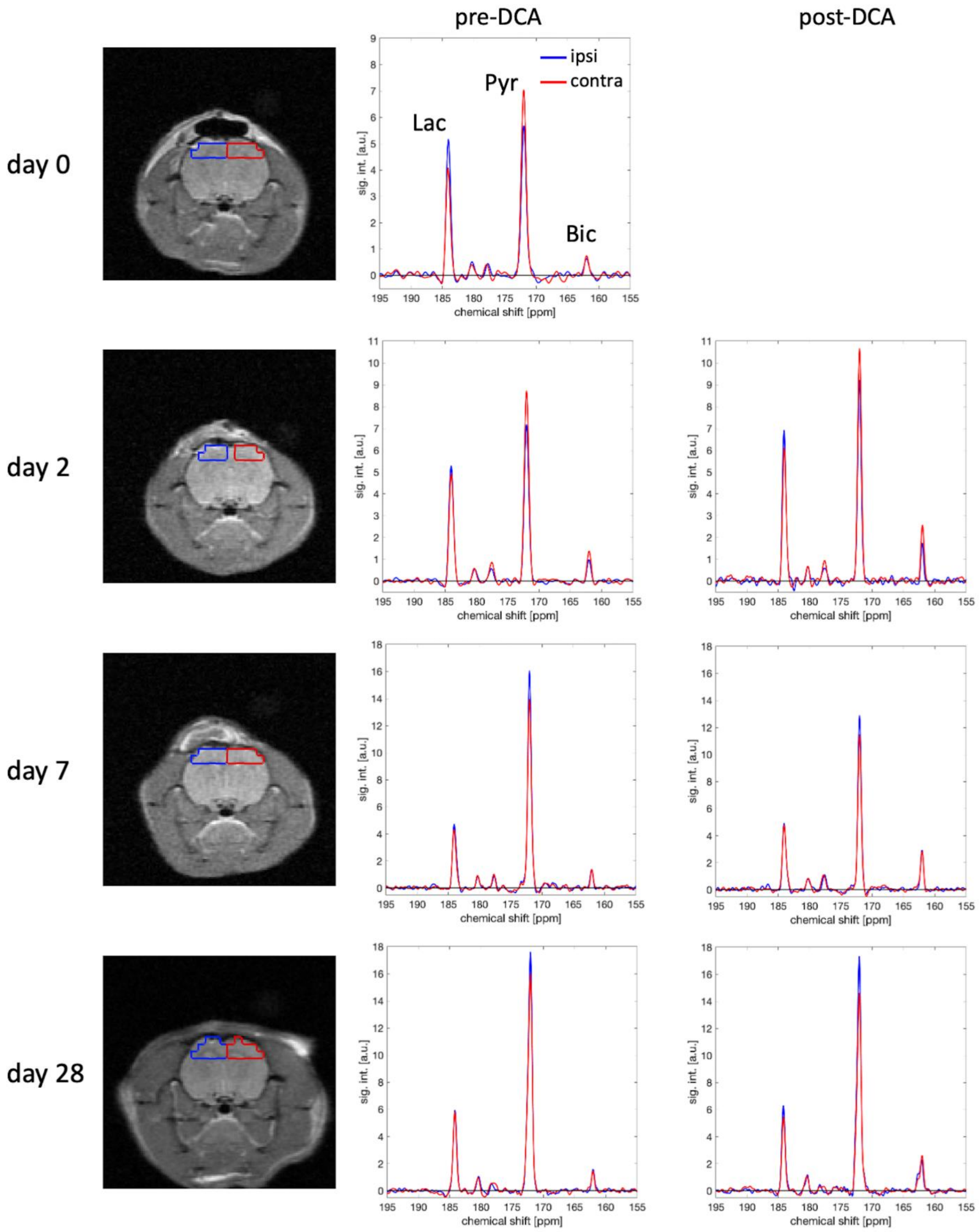

Figure S3. Longitudinal set of spectra in a CCI rat from ROIs in the ipsilateral (blue) and contralateral (red) hemisphere. The location of the ROIs is indicated on the corresponding MRIs (left). Spectra are shown both before (middle) and after (right) DCA administration. The same rat was imaged on days 0 through 28. No DCA was administered on day 0.

Table S1: Results of Welch's *t*-test for four different comparisons of bicarbonate concentration and bicarbonate-to-lactate ratio.

	**Bicarbonate**				**Bicarbonate/Lactate**			
	Ipsi vs. Contra		Ipsi/Contra vs.		Ipsi vs. Contra		Ipsi/Contra vs.	
Day	independent	correlated	control	sham	independent	correlated	control	sham
0	0.18	**0.042**	**0.018**	**0.049**	0.07	**0.026**	**0.0039**	**0.024**
2	0.21	**0.031**	**0.0004**	0.095	0.14	**0.008**	**<0.0001**	0.28
7	**0.013**	**0.041**	**0.028**	**0.032**	0.09	**0.013**	**0.008**	0.09
28	0.87	0.44	0.44		1.00	1.00	0.27	
