# Supplementary figures and images for: Enhancing Metabolic Imaging of Energy Metabolism in Traumatic Brain Injury Using Hyperpolarized [1-13C]Pyruvate and Dichloroacetate
Source: Metabolites. 2021 May 24;11(6):335. doi: 10.3390/metabo11060335 (PMC8225170; doi:10.3390/metabo11060335)

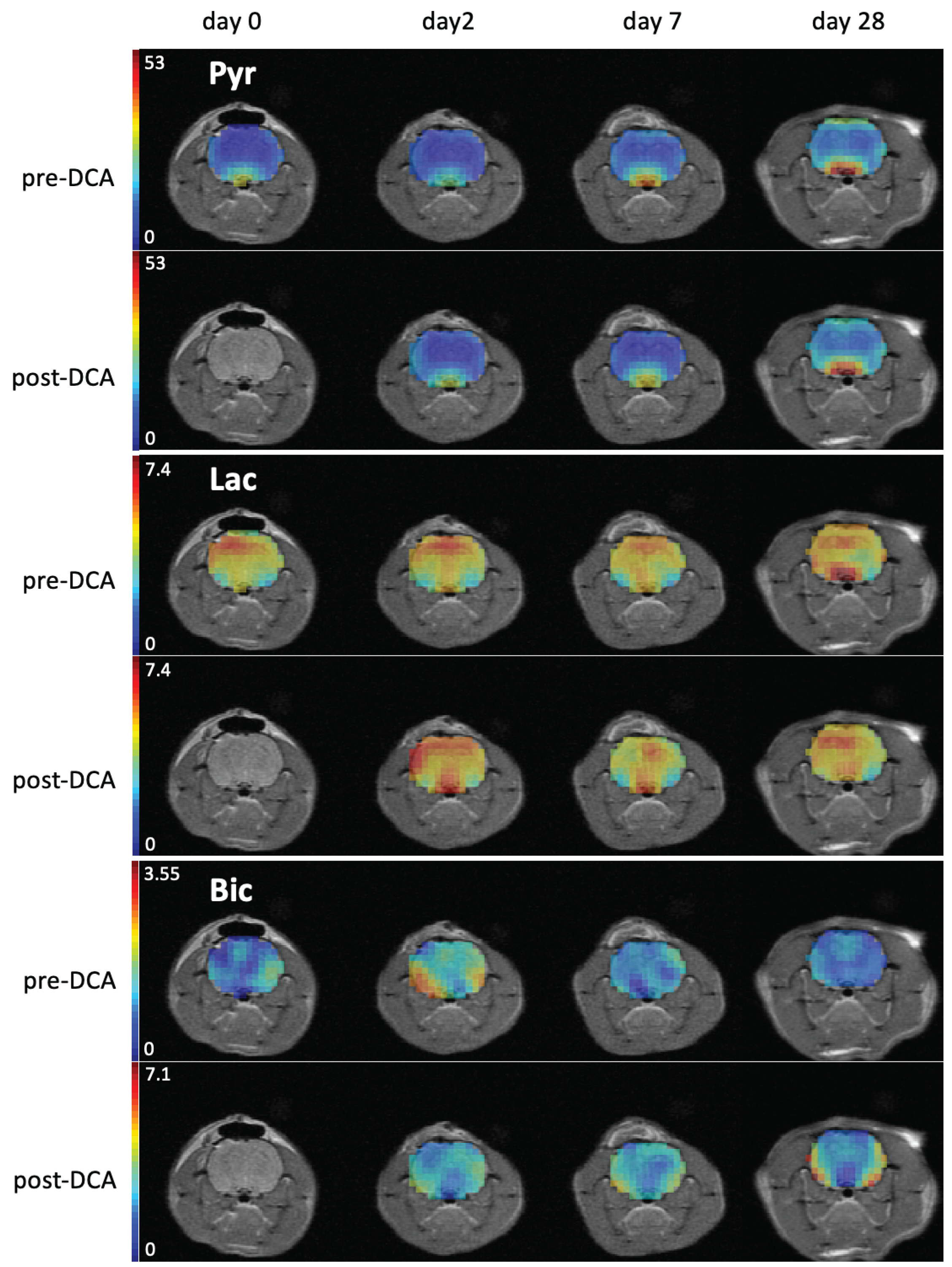

Supplement: Supplementary file 1 [file metabolites-11-00335-s001.zip › suppl/Supp_Fig1_rev.tif]

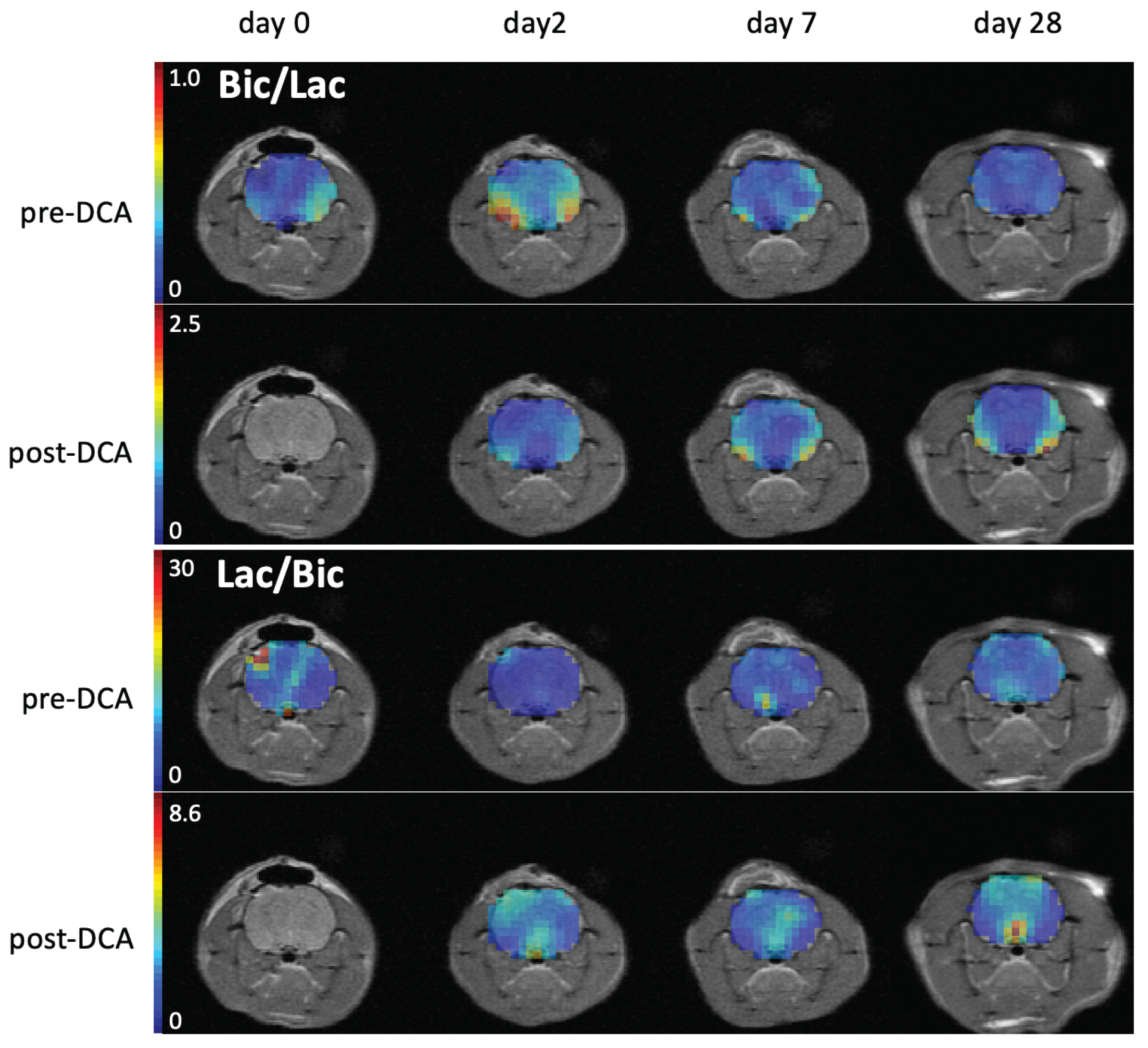

Supplement: Supplementary file 1 [file metabolites-11-00335-s001.zip › suppl/Supp_Fig2_rev.tif]

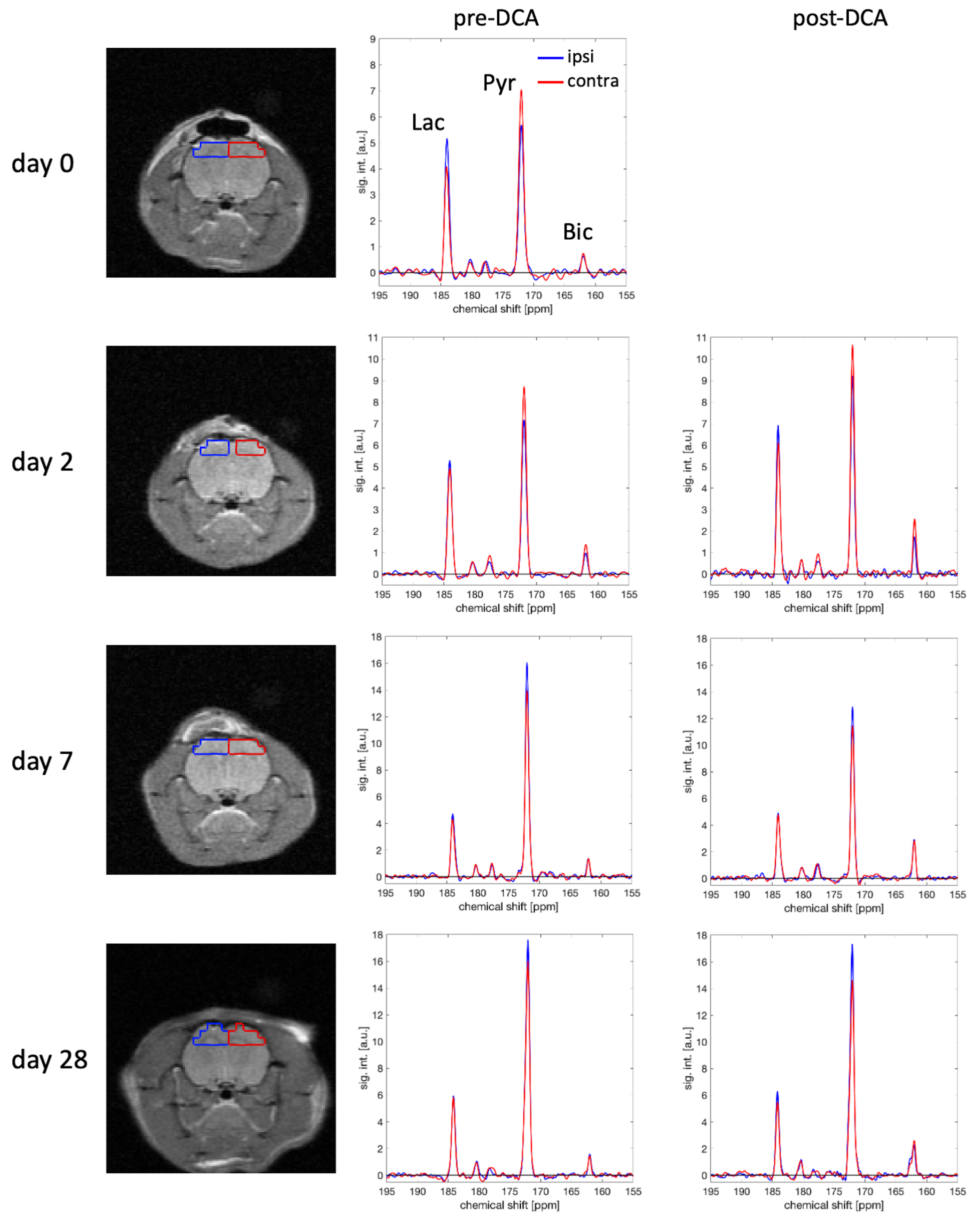

Supplement: Supplementary file 1 [file metabolites-11-00335-s001.zip › suppl/Supp_Fig3_rev.tif]
